# A Case of Colles Fracture with Luxation of the Ulna

**Published:** 1873-10

**Authors:** Charles D. Buckley

**Affiliations:** Rochester, N. Y.


ART. IV.—*A case of Colles Fracture with Luxation of the Ulna.* By CHAS. BUCKLEY, M. D., Rochester, N. Y.

Miss Agnes——, aged 20 years, residing in this city was employed in Kimball & Co's. Tobacco Factory.

On the 22d of January, 1872, she was engaged in attending a machine for packing tobacco in bags; part of this machine consists of a lever which descends and ascends to and from a table. While engaged in preparing bags for packing, her attention was called to another part of the building, at this time she was resting the lower end of the humerus on the table, the forearm being flexed on the arm and the radial border of the hand upturned, when in this position the lever descended on the hand, producing a colles fracture and a *compound luxation* of the ulna. This case is an interesting one in consequence of the luxatiou being compound instead of simple as usually occurs.

The appearance of the ulna was precisely the same as described by Dr. Moore, in an article read by him at the meeting of the New York State Medical Society, in 1870.

The carpal end of the ulna projected through the integument and internal annular ligament three-fourths of an inch, having the triangular fibro cartilage torn off.

The opening through the ligament resembled the external abdominal ring, its fibres being separated and firmly grasping the projecting end of the ulna.

Ether was administered and reduction accomplished with an audible snap which was remarked by the by-standers and Dr. Moore's dressing applied.

